# Loss of Atoh8 Impairs Macroautophagy

**DOI:** 10.3390/cells14241993

**Published:** 2025-12-15

**Authors:** Satya Srirama Karthik Divvela, Eric Bekoe Offei, Hawi Kadr, Maximilian Hausherr, Britta Eggers, Svitlana Rozanova, Martin Eisenacher, Hoang Duy Nguyen, Tran Tuoc, Verian Bader, Xuesong Yang, Holm Zaehres, Anqi Chen, Huu Phuc Nguyen, Konstanze F. Winklhofer, Katrin Marcus, Beate Brand-Saberi

**Affiliations:** 1 Department of Anatomy and Molecular Embryology, Medical Faculty, Ruhr University Bochum, 44801 Bochum, Germany; satya.divvela@rub.de (S.S.K.D.); eboffei@ug.edu.gh (E.B.O.); hawi.kadr@rub.de (H.K.); hoang.nguyenj79@ruhr-uni-bochum.de (H.D.N.); holm.zaehres@rub.de (H.Z.); 2 School of Veterinary Medicine, University of Ghana, Legon, Accra P.O. Box LG 68, Ghana; 3 Medizinisches Proteom-Center, Medical Faculty, Ruhr University Bochum, 44801 Bochum, Germany; maximilian.hausherr@rub.de (M.H.); britta.eggers@ruhr-uni-bochum.de (B.E.); svitlana.rozanova@rub.de (S.R.); martin.eisenacher@ruhr-uni-bochum.de (M.E.); katrin.marcus@rub.de (K.M.); 4 Medical Proteome Analysis, Center for Protein Diagnostics (PRODI), Ruhr University Bochum, 44801 Bochum, Germany; 5 Core Unit for Bioinformatics (CUBiMed.RUB), Medical Faculty, Ruhr University Bochum, 44801 Bochum, Germany; 6 Department of Human Genetics, Ruhr University Bochum, 44801 Bochum, Germany; tran.tuoc@ruhr-uni-bochum.de (T.T.); huu.nguyenr7w@rub.de (H.P.N.); 7 Department of Molecular Cell Biology, Institute of Biochemistry and Pathobiochemistry, Ruhr University Bochum, 44801 Bochum, Germany; verian.bader@rub.de (V.B.); konstanze.winklhofer@rub.de (K.F.W.); 8 Division of Histology and Embryology, Medical College, Jinan University, No 601 Huangpu Road West, Guangzhou 510632, China; yang_xuesong@126.com; 9 Institute of Forensic Science, Fudan University, Shanghai 200032, China; anqi_chen@fudan.edu.cn

**Keywords:** autophagy, macroautophagy, lysosome, Atoh8, p62, Tfeb, metabolism

## Abstract

The basic helix-loop-helix (bHLH) transcription factor ‘Atoh8’ is involved in the regulation of several developmental processes and pathologies. It regulates organogenesis, reprogramming, stem cell fate determination, and cancer development. However, the mechanisms underlying these observations remain unclear. Unlike many tissue-specific bHLH factors, Atoh8 is ubiquitously expressed during development as well as in adult tissues. In this study, we explored whether Atoh8 modulates basic cellular functions, which may reveal a common mechanism that could explain the diverse observations reported in the literature. Our findings demonstrate that the loss of Atoh8 impairs autophagy. In both primary myoblasts and mouse embryonic stem cells lacking Atoh8, we observed differential expression of LC3B-II, TFEB, and accumulation of p62, indicating impairment of autophagy. Furthermore, mass spectrometric analysis performed on C2C12 and Atoh8 overexpressing C2C12 myoblasts revealed significant alterations in the expression of proteins associated with mitochondrial and lysosomal functions. Finally, Cut&Tag sequencing performed in Atoh8 overexpressing C2C12 cells revealed that Atoh8 binds to multiple genes involved in autophagosome assembly. Overall, this study underscores that Atoh8 is a critical regulator of macroautophagy, and its reduction disrupts the autophagic process, whereas its overexpression results in increased autophagic flux.

## 1. Introduction

Atoh8 (Atonal homolog 8)/Math6 (Murine atonal homolog 6), a distant mammalian homolog of the Drosophila pro-neural gene ‘*atonal*’, encodes a basic helix-loop-helix (bHLH) transcription factor. Atoh8 plays diverse roles in multiple organ systems during embryonic development. Insights from mouse models suggest that Atoh8 regulates multiple processes, including neurogenesis, skeletal muscle development, pancreatic endocrine cell regulation, placental morphogenesis, and stem cell fate. Atoh8 was first identified in the context of neurogenesis, and its expression was detected in neuronal precursor cells of the ventricular zone and a subset of differentiating and mature neurons. Misexpression of Atoh8 in the developing retina supports neurogenesis and compromises gliogenesis [1]. It was further shown to be activated by BMP14, influencing the differentiation of retinal neurons, as the absence of Atoh8 impedes retinal stem cell differentiation [2]. Recently, Atoh8 has been shown to be a postnatal positive regulator of neurogenesis, modulating neuroblast proliferation, differentiation, and maintenance of mature neurons [3]. During skeletal myogenesis, it regulates myoblast proliferation. Absence of Atoh8 leads to reduced muscle mass, smaller fiber size, and atrophy [4]. In the developing pancreas, Atoh8 is expressed by endocrine progenitors. Conditional knockout mice (*Atoh8* Δpanc) demonstrated a modest increase in δ-cells (somatostatin-producing) without affecting the number of α- or β-cells, suggesting a selective role in δ-cell specification [5,6]. Furthermore, Atoh8 has been found to be critical for placental development. Global Atoh8 knockout mice exhibit mid-gestational hemorrhagic crises and fetal loss due to abnormal placental vasculature and decidualization [7]. In the context of stemness and pluripotency, murine embryonic stem cells lacking Atoh8 showed increased expression of core pluripotent factors Oct4 and Nanog and exhibited a propensity to differentiate into mesendoderm [8].

Recently, Atoh8 has gained attention in cancer research. It has been shown to play a dual role, functioning as both a tumor suppressor and promoter depending on the context and tissue type. In Non-small cell lung cancer (NSCLC), it acts as a tumor suppressor, inducing senescence via SMAD3 interaction and repressing cell cycle regulators such as CDK1 and CCNE2. The downregulation of Atoh8 has been correlated with aggressive phenotypes and therapy resistance [9]. Similarly, Atoh8 has been described as an intermediary in CYP3A5-driven suppression of lung adenocarcinoma metastasis by modulating SMAD1 phosphorylation [10]. In hepatocellular carcinoma (HCC), Atoh8 expression was consistently downregulated in tumor tissues compared to adjacent non-tumor tissues. Depletion of Atoh8 expression has been shown to correlate with advanced cancer stages and poor overall survival. Atoh8 depletion also increases the expression of cancer stem cell markers, such as OCT4, NANOG, and CD133. Atoh8 overexpression has been attributed to improved chemosensitivity and modulation of the tumor microenvironment [11,12]. Similarly, Atoh8 has been observed to be downregulated in nasopharyngeal carcinoma (NPC) tissues and derived cell lines. Lower levels of Atoh8 have been associated with advanced tumor stage, metastasis, and poor survival [13]. In contrast to its tumor-suppressive function in the above-mentioned cancer types, Atoh8 has been identified to be upregulated in colorectal cancer and is correlated with reduced median overall survival. Inhibition of Atoh8 in SW620 cells resulted in decreased proliferation and increased chemosensitivity. The functional duality of Atoh8 is attributed to the AKT pathway, as it regulates tissue-dependent activation or inhibition of the AKT pathway [12,14].

In addition to its role in embryonic development and carcinogenesis, it has emerged as a regulator of several metabolic processes. Atoh8 has been characterized for its role in iron metabolism. Atoh8 regulates hepcidin (HAMP), a key hormone that prevents iron overload. Atoh8 expression levels were found to be reduced in liver tissues under conditions of high erythropoietic activity, such as hypoxia, hemolytic anemia, hypotransferrinemia, and erythropoietin treatment. Conversely, Atoh8 expression was observed to increase following treatment with erythropoietic inhibitors [15]. Regarding glucose metabolism, conditional pancreatic Atoh8 knockout mice showed an increase in the δ-cell compartment of the pancreas, with a modest reduction in glucose and insulin levels [6]. In the context of glucose metabolism and pancreatic function, Atoh8 has been identified as a downstream factor of ATF4, a regulator of the unfolded protein response (UPR) and integrated stress response (ISR). Loss of Atf4 in pancreatic cells results in depletion of Atoh8 and upregulation of dedifferentiation markers such as OCT4 and NANOG [16]. Atoh8 has also been shown to regulate cellular responses to hypoxia. Atoh8 was found to directly interact with hypoxia-inducible factor 2α (HIF-2α) and decrease its abundance, attenuating the HIF-2α dependent hypoxic response. Atoh8 knockout mice generated by the same group showed increased pulmonary arterial pressure and right ventricular hypertrophy [17]. Consistent with these findings, our previous work demonstrated that the loss of Atoh8 leads to an increase in oxidative myofibers at the expense of fast glycolytic myofibers [4].

The resemblance of phenotypes in Atoh8 knockout mice to those seen in autophagy-deficient models such as impaired placental development and fetal mortality [7,18], combined with Atoh8’s roles in cell reprogramming and stress responses [8,17,19,20,21,22], suggests a possible functional link between Atoh8 and autophagy that remains unexplored. Macroautophagy, commonly referred to as autophagy, is a highly conserved cellular degradation process employed by cells to degrade cytoplasmic contents and recycle or eliminate redundant organelles and harmful molecules [23,24]. The main functions of autophagy include energy production, provision of metabolic precursors necessary for energy generation, provision of macromolecules for macromolecular biosynthesis [25], and intracellular clearance [26]. The main cellular autophagy machinery has been described in six (6) distinct steps: induction, phagophore nucleation, membrane elongation and cargo sequestration, membrane closure and maturation, lysosomal fusion, and degradation [27,28].

In the present study, we investigated the effects of the loss- and gain-of-function of Atoh8 on autophagy and showed that Atoh8 regulates autophagy.

## 2. Materials and Methods

### 2.1. Isolation, Culture of Mouse Primary Myoblasts and C2C12 Myoblasts

Owing to the absence of a highly specific anti-Atoh8 antibody designed to detect the murine Atoh8 protein, we generated *Atoh8^Flag-tag^* mice to serve as wildtype (WT) [8] and Atoh8 knockout (*Atoh8^−/−^* or KO) mice [7]. Both mouse types used in the study were of the ‘C57Bl/6NJ’ strain. Primary myoblasts were isolated as previously described [29]. Mouse primary myoblasts were cultured in high-glucose DMEM (Pan-Biotech, Aidenbach, Germany) supplemented with 20% fetal bovine serum (Pan-Biotech), 1% L-glutamine (Gibco, Grand Island, NY, USA), 1% non-essential aminoacids (NEAA) (Gibco), 1% Penicillin-Streptomycin (P/S) (Gibco), and 10 µg/mL bFGF (Peprotech, Cranbury, NJ, USA). C2C12 myoblasts were cultured in high-glucose DMEM (Pan-Biotech) supplemented with 10% fetal bovine serum (Pan-Biotech), 1% L-glutamine (Gibco), 1% NEAA (Gibco), and 1% P/S (Gibco).

### 2.2. Mouse Embryonic Stem Cell Culture

The mouse embryonic stem cell lines used in this study, namely the WT and KO mouse embryonic stem cell lines, have been described previously [8]. Blastocysts from *Atoh8^Flag-tag^* and *Atoh8^−/−^* mice were isolated and embryonic stem cells (ESCs) were established using the protocol described by Brownstein [30]. ESCs were cultured under serum-free conditions in 2i/LIF medium. The media composition used is as follows: 50% Neurobasal medium (Pan-Biotech), 50% DMEM/F12 (Pan-Biotech), N2 supplement (1:200) (Gibco), B27 supplement (1:100) (Gibco), 1% penicillin-streptomycin, 1% L-glutamine, LIF (1000 U/mL) (Peprotech, Cranbury, NJ, USA), PD0325901 (1 μM) Peprotech, Cranbury, NJ, USA), and CHIR99021 (3 μM) Peprotech, Cranbury, NJ, USA). The cells were cultured in 4% Matrigel-coated plates and passaged every 96 h at a seeding density of 1000 cells/cm^2^.

### 2.3. Generation of Atoh8-Flag Overexpressing C2C12 Myoblasts and GFP-LC3-RFP Labeled WT and KO Primary Myoblasts

To create *Atoh8^Flagtag^* overexpressing stable C2C12 cells (C2C12-Atoh8-OE), we transduced C2C12 myoblasts with a retrovirus containing the *Atoh8-Flag* sequence. The virus was produced using HEK cells, following a previously established protocol [8]. C2C12 myoblasts were infected with 50 µL of virus twice after 48 and 72 h of transfection, with each infection occurring on consecutive days and lasting for 4 h. Similarly, to study autophagic flux, both WT and KO primary myoblasts were infected with a virus carrying ‘GFP-LC3-RFP,’ as mentioned above. The pMXs-GFP-LC3-RFP reporter plasmid was obtained from Addgene (Watertown, MA, USA) (117413) [31].

### 2.4. Western Blot

The cell cultures were washed once with ice-cold 1× PBS and subsequently lysed using RIPA buffer supplemented with a protease inhibitor cocktail at a 1:100 dilution ratio. Protein concentration was determined using Bradford’s protein quantification method. A total of 50 µg of protein was loaded onto the SDS-PAGE gel, and electrophoresis was conducted at 130 V for 60 min at room temperature. Following SDS-PAGE electrophoresis, proteins were transferred from the gel to a nitrocellulose membrane via wet blotting for 90 min at 200 mA at 8 °C. After transfer, the nitrocellulose membrane was blocked with a 10% blocking solution composed of non-fat dry milk powder for 2 h. Subsequently, the blots were incubated with the appropriate primary antibody at 4 °C overnight, followed by incubation with secondary antibodies conjugated to horseradish peroxidase (HRP) (1:1000) at room temperature for one hour. To detect Atoh8-Flag protein in primary myoblasts and ESCs, cells were treated with MG132 (10 μM) for 6 h prior to preparation of cell lysates. MG132 treatment was performed to improve the detection of Atoh8-Flag protein as its half-life is approximately 30 min, which was also previously reported [17]. Imaging was performed using ECL reagent (Bio-Rad, Hercules, CA, USA, 1705060). Antibodies used in the study are as follows: LC3B (Novus-NB100-2220 (1:1000), Centennial, CO, USA); P62 (R&D-MAB8028 (1:1000), Minneapolis, MN, USA, P62 (Cell Signaling Technology-5114S (1:1000), Danvers, MA, USA); TFEB (Proteintech-13372-1-AP (1:1000), Rosemont, IL, USA); Anti-Flag (Sigma-Aldrich-F1804 (1:1000), St. Louis, MO, USA).

### 2.5. Immunostaining

Cells cultured in 24-well plates were subjected to two washes with 1× PBS and subsequently fixed with 4% paraformaldehyde (PFA) for 15 min. Following fixation, the cells were permeabilized using 0.5% Triton X-100 for 10 min and then blocked with 5% bovine serum albumin (BSA; Life Technologies, Carlsbad, CA, USA) for 1 h at room temperature. After blocking, the cells were incubated with the TFEB primary antibody (Proteintech-13372-1-AP) at 4 °C overnight. The cells were then washed three times with 1× PBS and incubated with the secondary antibody for 1 h at room temperature (Invitrogen-A11011, Waltham, MA, USA). Finally, the cells were washed thrice with 1× PBS and mounted using a mounting medium containing DAPI (Invitrogen, S36920). HEK cells were cultured for 72 h after transfection (Atoh8-KD and Atoh8-OE) and stained with an anti-TFEB antibody. To quantify the subcellular localization of TFEB, a pipeline from cell profiler was used, with minor modifications, to detect the red signal [32]. Regarding GFP-LC3-RFP labeled primary myoblasts, the cells underwent two washes with 1× PBS and were subsequently fixed with 4% paraformaldehyde (PFA) for 15 min. Following fixation, the coverslips were mounted on slides using a mounting medium containing DAPI (Invitrogen, S36920). The cells were imaged using the Zeiss LSM 800. Images of GFP-LC3-labeled myoblasts were converted to an 8-bit format, and autophagosomes were subsequently quantified using the ‘Analyzer particle’ tool in Fiji (version 1.54p) [33].

### 2.6. Animal Handling

The study adhered to the European Communities Council Directive of 2010 (2010/63/EU) for animal welfare in laboratory experiments and was authorized by the Animal Care Committee of North Rhine-Westphalia, Germany, located at the Landesamt für Umweltschutz, Naturschutz und Verbraucherschutz (LANUV). Nordrhein-Westfalen, D-45659, Recklinghausen, Germany). The Animal Welfare Commission of Ruhr University Bochum oversaw this study. Efforts were made to reduce the number of mice to the maximum possible level. The mice used in this study were bred in-house and maintained on a 12 h dark/light cycle with food and water ad libitum.

### 2.7. Cut&Tag Sequencing

The CUT&Tag-IT^®^ Express Assay Kit (Active Motif #53175, Carlsbad, CA, USA) was used, with some modifications. After cell collection, cell density was calculated and normalized to a concentration of approximately 177,000 cells per sample. The Concanavalin A beads binding cells were incubated with 1 µg primary antibodies, for example, FLAG antibody (Sigma-Aldrich, F1804) (n = 4) as the targeted group, normal IgG (Cell Signaling Technology, #2729) (n = 4) as the negative control group, and H3K27ac antibody (Abcam, Cambridge, UK, ab4729) (n = 1) as the positive control group. The next day, the compensated secondary antibody for each reaction was applied. Next, CUT&Tag-IT™ assembled pA-Tn5 transpososomes were added. After Tagmentation, DNA was extracted and collected using silica beads before it was amplified with a combination of indexed primers. Library was collected with SPRI Beads (Beckman Coulter, Brea, CA, USA, # B23318).

### 2.8. Cut&Tag Sequencing Data Analysis

The data analysis for Cut&Tag Sequencing was based on the protocol of Zheng Y et al. (2020) [34]. ChIPseqSpikeInFree was used instead of Spike-In calibration [35]. The peak calling was performed using MACS3 (version 3.0.2) [36]. Differential expression analysis was supported by DESeq2 (version 1.42.1) [37]. This paper does not report any original code. Any additional information required to reanalyze the data reported in this paper is available from the lead contact upon request.

### 2.9. Cell Lysis and Digestion

C2C12 and Atoh8 overexpressing cell pellets were lysed as described previously, with slight modifications (Prisacar 2025 [38]). In brief, cells were lysed in DIGE buffer (7 M Urea, 2 M Thiourea, 30 mM Tris Base, pH 8), supported by mechanical lysis utilizing a pestle. To shear DNA, the lysates were sonicated in a sonication bath three times for 5 min with a resting period on ice between each sonication cycle. Lysates were centrifuged for 10 min at 15,000× *g* at 4 °C, and the resulting supernatant was transferred to another vial. Protein concentration was determined by Bradford assay and 20 µg of protein was digested in solution. Prior to digestion, proteins were reduced by adding DTT (final concentration 5 mM) at 56 °C for 30 min and alkylated with IAA (final concentration 15 mM) for 30 min at room temperature. Digestion was carried out overnight using trypsin (ratio 1:40). Digestion was stopped by acidification with trifluoroacetic acid (final concentration 0.5%).

### 2.10. Liquid Chromatography and Mass Spectrometry

Liquid chromatography was performed on an Evosep One system (Evosep, Odense, Denmark) online coupled to a Orbitrap Exploris 480 mass spectrometer (ThermoFisher Scientific, Bremen, Germany), equipped with a nanoelectrospray ionization source with a steel emitter for ion injection. Each sample was loaded onto individual Evotips for desalting according to the manufacturer’s protocol. Briefly, the Evotips were washed with buffer B (99.9% ACN, 0.1% FA), activated with isopropanol, and equilibrated with buffer A (99.9% H_2_O, 0.1% FA). In between, the Evotips were centrifuged at 80× *g* for 1 min. Then, 20 µL of sample containing 100 ng of peptides were loaded, centrifuged, and washed with 20 µL buffer A. After loading and washing, the Evotips were centrifuged at 800× *g* for 1 min. For storage, 100 μL buffer A was applied on the tip, centrifuged at 800× *g* for 10 s, to keep the Evotip wet until analysis. Peptide separation was performed with the Evosep One system (Evosep, Odense, Denmark) utilizing a Evosep Performance column (PepSep C18, 15 cm × 75 µm × 1.9 µm) (EvoSep, Denmark) at 40 °C with the 30 SPD (samples per day) method.

Samples were measured in data independent acquisition mode (DIA). MS1 scans were carried out in positive scan mode, whereby a scan range of 350–1000 *m*/*z* and an orbitrap resolution of 120,000 were chosen. For both MS1 and MS2 scans an RF lens of 50% was deployed, the AGC target was set to standard and the maximum injection time was automatically chosen by the instrument. On the MS2 level, 16 windows with a window size of 32 *m*/*z* spanning 400–900 *m*/*z* were added. Fragment ions were induced by higher energy collisional dissociation (HCD) with a normalized collision energy of 31% and scanned with an orbitrap resolution of 30,000. The resulting raw data was analyzed in Spectronaut (version 20.2.250922.92449, Biognoysis, Schlieren, Switzerland), utilizing the direct DIA option. BGS factory settings were chosen, with the exception of adding trypsin as digestion enzyme and carbamidomethylation on cysteines as fixed and oxidation on methionine as variable modification. Spectra were searched against the mus musculus fasta obtained from uniprot (54,739 entries, “uniprot.org (accessed 28 February 2024)”. For quantification the proteotypicity filter was set to protein group specific. Normalization was disabled and carried out externally. The mass spectrometry proteomics data have been deposited to the Proteome-Xchange Consortium via the PRIDE partner repository with the dataset identifier PXD070896 [39,40].

The protein group quantities obtained from Spectronaut were LOESS-normalized and analyzed further using R (version 4.5.0, R Core Team, 2021, https://www.R-project.org/ (accessed on 10 November 2025)) with the ‘ProtStatsWF’ package (version 1.0.0, https://github.com/mpc-bioinformatics/ProtStatsWF (accessed on 10 November 2025)). The quality of the normalization was evaluated using boxplots and MA plots. Further inter- and intra-group differences were studied and visualized using principal component analysis (PCA) plots based on LOESS-normalized log_2_ quantities. Only proteins without missing quantity values were included in the PCA and further differential analysis. For the differentiation analysis of protein levels in the two corresponding conditions a two-sided Welch’s *t*-test with Benjamini–Hochberg FDR correction of *p*-values (q-values) was applied. Proteins with q-values < 0.05 were considered significantly differentially abundant between the two conditions. The *t*-test results were visualized using R version 4.5.0 (R Core Team, 2021) and the ‘EnhancedVolcano’ package version 1.22.0 [https://github.com/kevinblighe/EnhancedVolcano (accessed on 10 November 2025)].

### 2.11. Statistical Analysis

To determine statistical significance, the data were handled as follows. All Western blot data were analyzed by performing *t*-tests using the Holm–Sidak method. For quantification of TFEB translocation, we performed unpaired, nonparametric, two-tailed, Kolmogorov–Smirnov D *t*-tests.

## 3. Results

### 3.1. The Loss of Atoh8 Leads to Downregulation of LC3B-II in Embryonic Stem Cells but Not in Differentiated Cells

**Figure 1:**
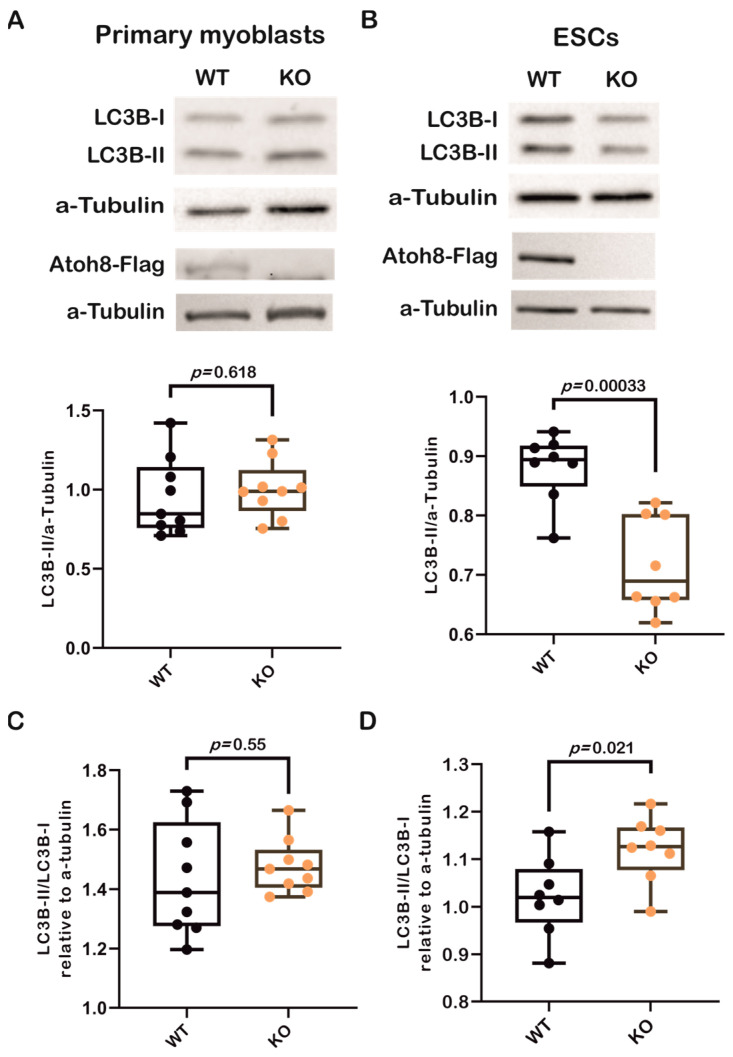
Loss of Atoh8 does not alter LC3B-II levels in terminally differentiated cells, except in ESCs. (**A**) Representative Western blot images (top) show LC3B-I, LC3B-II, and a-Tubulin in wildtype (WT) and Atoh8 knockout (KO) primary myoblasts. The corresponding box plot (bottom) shows the LC3B-II/a-Tubulin ratios in WT and KO primary myoblasts. No significant difference was observed between the WT and KO samples (*p* = 0.618), (n = 9). Atoh8-Flag protein expression is shown below LC3, Western blot image showing Atoh8-Flag expression in WT whereas no expression is seen in KO primary myoblasts. (**B**) Western blot images (top) display LC3B-I, LC3B-II, and α-Tubulin in WT- and KO-ESCs. The box plot (bottom) shows LC3B-II/a-Tubulin ratios; and a significant reduction in LC3B-II levels in KO-ESCs (KO) compared to WT-ESCs (WT) (*p* = 0.00033), (n = 8). Atoh8-Flag protein expression is shown below LC3, Western blot image showing Atoh8-Flag expression in WT whereas no expression is seen in KO-ESCs. (**C**) Box plots show a comparison of LC3B-II/LC3B-I ratio between wildtype (WT) and knockout (KO) primary myoblasts. No significant difference was observed (*p* = 0.55) in the LC3B-II/LC3B-I ratio relative to a-tubulin between WT and KO cells (n = 9). (**D**). Box plots show a comparison of LC3B-II/LC3B-I ratio between WT- and KO-ESCs. KO-ESCs showed a statistically significant increase in the LC3B-II/LC3B-I ratio compared to WT-ESCs (*p* = 0.021) (n = 8).

First, we analyzed LC3B-II expression in primary myoblasts derived from wildtype (WT) and Atoh8 knockout (KO) mice and did not observe any significant difference in the expression of LC3B-II between WT and KO myoblasts (WT vs. KO; *p* = 0.618) (Figure 1A). In addition to terminally differentiated cells, we next analyzed the expression of LC3B-II in mouse embryonic stem cells (ESCs). Surprisingly, we observed a significant downregulation of LC3B-II in KO-ESCs compared to that in WT-ESCs (*p* < 0.00033) (Figure 1B). To confirm these findings, we further evaluated the ratio of LC3B-II (lipidated) and LC3B-I (nonlipidated) forms to determine autophagic activity in these cells. We observed tendency of high LC3B-II/LC3B-I ratio in KO primary myoblasts (WT vs. KO; *p* = 0.55) and a significant increase in the LC3B-II/LC3B-I ratio in KO-ESCs (WT vs. KO; *p* = 0.021) (Figure 1C,D), suggesting altered autophagic activity in KO cells compared to WT controls. Collectively, the data indicate that the absence of Atoh8 appears to result in a modified autophagic activity in primary myoblasts and ESCs, as evidenced by both LC3B-II expression and the LC3B-II/LC3B-I ratio.

### 3.2. Loss of Atoh8 Results in the Accumulation of p62 Under Nutrient-Rich Conditions

**Figure 2:**
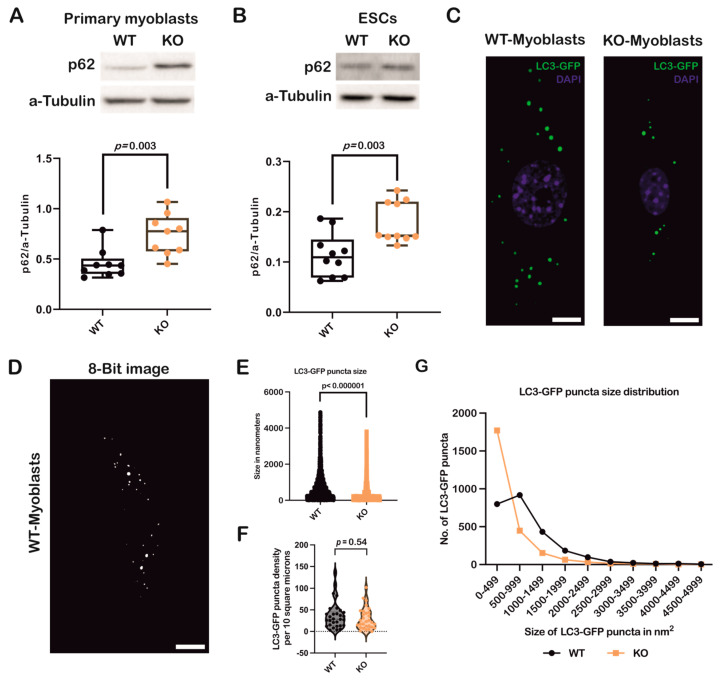
Loss of Atoh8 results in upregulation/accumulation of p62 impairing autophagy. (**A**) Western blot images (top) show p62 and a-Tubulin in wildtype (WT) and Atoh8 knockout (KO) primary myoblasts. The corresponding box plot (bottom) shows the p62/a-Tubulin ratios in WT and KO primary myoblasts. A significant difference was observed between WT and KO primary myoblasts (*p* = 0.003) (n = 9). (**B**) Western blot images (top) display p62 and a-Tubulin in WT- and KO-ESCs. The box plot (bottom) shows p62/a-Tubulin ratios; and a significant increase in p62 levels in KO-ESCs (KO) compared to WT-ESCs (WT) (*p* = 0.003) (n = 10). (**C**) Fluorescence images depict GFP-LC3 labeled autophagic puncta in wild-type (WT) and knockout (KO) primary myoblasts. Scale bar represents 10 µm. (**D**) A representative image of WT primary myoblasts, which was converted into an 8-bit format and subsequently adjusted to a threshold to eliminate background noise. These processed images were then used to quantify the number and size of autophagosomes using Fiji (version 1.54p) Scale bar represents 10 µm. (**E**) A comparison of LC3GFP puncta sizes between WT and KO primary myoblasts revealed that KO primary myoblasts exhibited significantly smaller autophagosomes than their WT counterparts (*p* < 0.000001) (n = 90). (**F**) A comparison of LC3-GFP puncta density between WT and KO primary myoblasts. No significant difference in the LC3-GFP puncta density per 10 square microns was observed between WT and KO primary myoblasts (*p* = 0.54). (**G**) Analysis of the size distribution of autophagosomes in WT and KO primary myoblasts indicated a significant difference in the size of autophagosomes in KO primary myoblasts. KO primary myoblasts demonstrated a significantly higher number of small autophagosomes than their WT counterparts.

To further confirm autophagic status with respect to Atoh8, we analyzed p62 expression in primary myoblasts and ESCs. Surprisingly, the analysis performed on WT and KO primary myoblasts showed a significant upregulation of p62 in KO primary myoblasts compared to WT primary myoblasts (WT vs. KO; *p* = 0.003) (Figure 2A). We further analyzed p62 expression in ESCs (WT vs. KO; *p* = 0.003) (Figure 2B), and observed a consistent and statistically significant upregulation of p62 following knockout of Atoh8. We then transduced WT and KO primary myoblasts with a retrovirus carrying the ‘GFP-LC3-RFP’ fusion protein and quantified the number of LC3-GFP puncta (Figure 2C,D). A comparison of LC3-GFP puncta size revealed a significant reduction in KO primary myoblasts compared to WT cells (WT vs. KO; *p* < 0.000001) (Figure 2E). However, the quantification of LC3-GFP puncta did not show any significant difference in the density of LC3-GFP puncta between WT and KO primary myoblasts (WT vs. KO; *p* = 0.54) (Figure 2F). We further analyzed the size distribution of LC3-GFP puncta in primary myoblasts. KO primary myoblasts exhibited a higher number of smaller LC3-GFP puncta than WT cells, suggesting impairment in either autophagosome formation and maturation in KO cells compared to WT cells (Figure 2G). Overall, these data demonstrate that the loss of Atoh8 impairs autophagy, highlighting its critical role in autophagosome maturation and degradation.

### 3.3. Stable Atoh8 Overexpression Resulted in the Downregulation of p62

**Figure 3:**
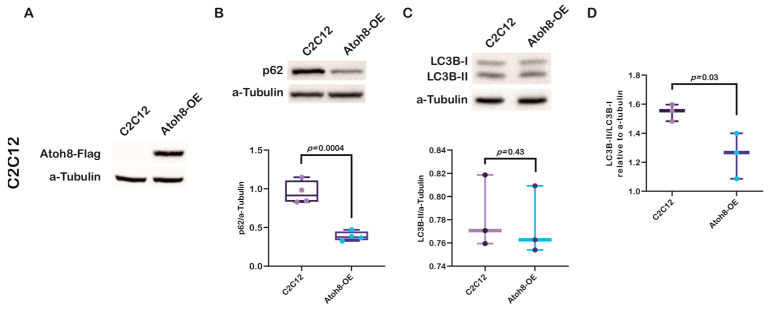
Stable Atoh8 overexpression results in downregulation of p62 expression in C2C12 cells. (**A**) Western blot images showing ATOH8-Flag expression in C2C12 and Atoh8-OE myoblasts. (**B**) Western blot images (top) and quantification (bottom) of p62 protein levels normalized to a-Tubulin in C2C12 myoblasts (C2C12) and C2C12 cells overexpressing Atoh8 (Atoh8-OE). Atoh8 overexpression significantly decreased p62 levels compared to that in the control (*p* = 0.0004) (n = 4). (**C**) Western blot images (top) and quantification (bottom) of LC3B-II protein levels normalized to a-Tubulin in C2C12 and Atoh8-OE cells. No significant difference in the LC3B-II/a-Tubulin ratio was observed between the groups (*p* = 0.43) (n = 3). (**D**) Comparison of LC3B-II/LC3B-I ratio between C2C12 and stable Atoh8 overexpressing C2C12 cells (Atoh8-OE) (n = 3). A significantly lower LC3B-II/LC3B-I ratio was observed in Atoh8 overexpressing C2C12 cells (*p* = 0.03) than in C2C12 cells.

To further confirm the role of Atoh8 in the regulation of autophagy, we generated a C2C12 myoblast cell line that overexpress *Atoh8-Flag* and evaluated the expression of p62 (Figure 3A). As expected, we observed a significant downregulation in the expression of p62 following Atoh8 overexpression compared to that in untransduced cells (C2C12 vs. Atoh8-OE; *p* = 0.0004) (Figure 3B). In addition, we evaluated the expression of LC3B-II and observed no difference in the expression of LC3B-II between C2C12 myoblasts and Atoh8 overexpressing C2C12 cells (C2C12 vs. Atoh8-OE; *p* = 0.43) (Figure 3C). These findings suggest that Atoh8 plays a role in autophagy-specific degradation. To further verify these findings, we evaluated the LC3B-II/LC3B-I ratio to determine autophagic activity. As expected, we observed a significant reduction in the LC3B-II/LC3B-I ratios following stable overexpression of Atoh8 in C2C12 cells (C2C12 vs. Atoh8-OE; *p* = 0.03) (Figure 3D). The expression of p62, together with the reduction in the LC3B-II/LC3B-I ratio following Atoh8 overexpression, suggests increased autophagic activity. However, in this regard to better understand the mechanistic role of Atoh8 in autophagy regulation, we decided to examine the master regulator of autophagosome formation and lysosomal biogenesis called ‘TFEB.’

### 3.4. Modulation of Autophagy by Atoh8 Does Not Directly Depend on TFEB

**Figure 4:**
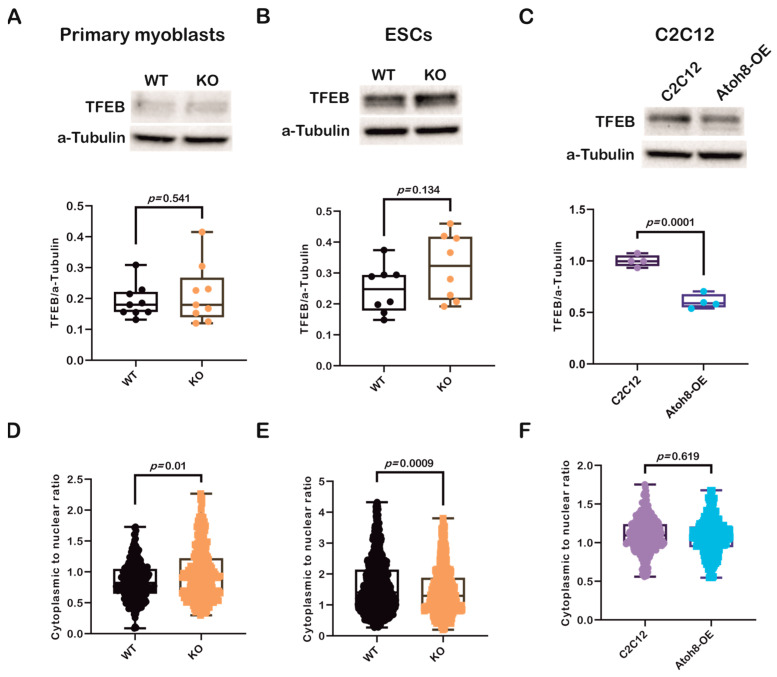
Modulation of autophagy by Atoh8 does not directly depend on TFEB. (**A**) Western blot images (Top) and quantification of TFEB protein levels (Bottom), normalized to a-Tubulin in WT and KO primary myoblasts. No significant difference in TFEB expression was observed between WT and KO (*p* = 0.541) (n = 9). (**B**) Western blot images (Top) and quantification of TFEB protein levels (Bottom), normalized to a-Tubulin in WT- and KO-ESCs. No significant difference in TFEB expression was observed between WT- and KO-ESCs (*p* = 0.134) (n = 8). (**C**) Western blot images (Top) and quantification of TFEB levels in C2C12 and Atoh8-OE cells (Bottom), showing a significant reduction in TFEB protein in Atoh8-OE cells (*p* = 0.0001) (n = 4). (**D**) Quantification of subcellular localization of TFEB, Violin plot showing the cytoplasmic to nuclear ratio of TFEB in WT and KO primary myoblasts. KO cells displayed a significantly higher cytoplasmic-to-nuclear TFEB ratio than WT cells (*p* = 0.01), indicating decreased nuclear localization (n = 250). (**E**) Violin plot showing the cytoplasmic to nuclear ratio of TFEB in WT- and KO-ESCs. KO-ESCs displayed a significantly lower cytoplasmic-to-nuclear TFEB ratio than WT (*p* = 0.0009), indicating increased nuclear localization (n = 650). (**F**) Violin plot showing cytoplasmic to nuclear TFEB ratio in C2C12 and Atoh8-OE cells, showing no significant difference (*p* = 0.619) (n = 250).

Transcription factor EB (TFEB) is a basic helix-loop-helix leucine zipper transcription factor that regulates macroautophagy and lysosomal biogenesis [41]. It regulates key genes involved in autophagy. It activates genes involved in the initiation of autophagosomal membranes (Beclin1, NRBF2, and ATG9B), elongation of the autophagosomal membrane (GABARAP), and fusion of autophagosomes and lysosomes (RAB7A, UVRAG, and MCOLN1) [42,43]. As TFEB also belongs to the bHLH superfamily of transcription factors, such as Atoh8, we further evaluated TFEB expression to investigate whether there was any correlation between these factors and their influence on autophagy. Contrary to our expectations, we did not observe any significant differences in TFEB expression between the WT and KO primary myoblasts (WT vs. KO; *p* = 0.479) (Figure 4A). Similarly, no difference was observed in expression between WT and KO-ESCs (WT vs. KO; *p* = 0.137) (Figure 4B). However, surprisingly, we observed a significant downregulation in C2C12-Atoh8-OE cells following stable overexpression of *Atoh8-Flag*, suggesting a negative correlation between these factors (C2C12 vs. C2C12-Atoh8-OE; *p* < 0.001) (Figure 4C). Furthermore, to determine the differential regulation of TFEB in different cell lines, we analyzed TFEB subcellular localization and compared its cytoplasmic and nuclear localization to better understand its dynamics with respect to the presence of Atoh8. We observed significant retention of Atoh8 in the cytoplasm of KO primary myoblasts compared to that in WT primary myoblasts (WT vs. KO; *p* = 0.01) (Figure 4D and Supplementary Figure S1). Contrary to observations in terminally differentiated cells (primary myoblasts), KO-ESCs showed increased nuclear expression of TFEB, suggesting an adaptive mechanism in pluripotent stem cells following the knockout of Atoh8 (WT vs. KO; *p* = 0.0009) (Figure 4E and Supplementary Figure S2). Next, we quantified TFEB localization following stable ATOH8 overexpression in the C2C12 cells. Surprisingly, we did not observe any significant difference in the cytoplasmic-to-nuclear ratio between C2C12 and C2C12-Atoh8-OE cells, despite the significant downregulation of total TFEB expression (C2C12 vs. Atoh8-OE; *p* = 0.619) (Figure 4F and Supplementary Figure S3). Altogether, these findings indicate that changes in total TFEB expression and its subcellular localization dynamics reflect a compensatory response to altered autophagic status caused by either the loss or overexpression of Atoh8.

### 3.5. Bafilomycin A1 Treatment Supports Atoh8’s Involvement in Autophagy

**Figure 5:**
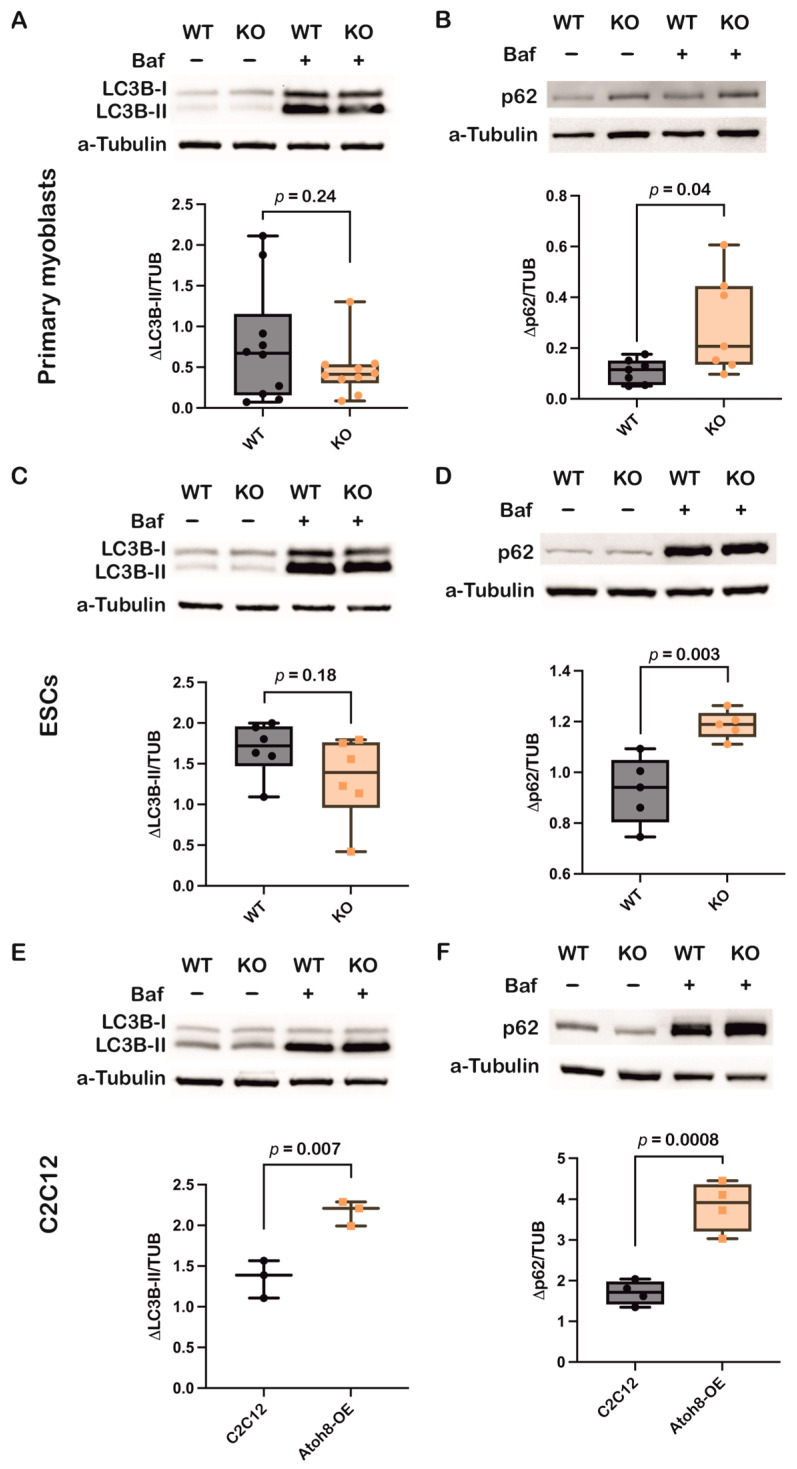
Inhibition of autophagy by Bafilomycin A1 confirms involvement of Atoh8 in the regulation of autophagy. (**A**) Western blot images showing LC3B-II protein levels in wildtype (WT) and knockout (KO) primary myoblasts, both under basal (Baf−) and bafilomycin A1-treated conditions (Baf+). a-tubulin was analyzed as loading control. Box plot shows no significant difference in ΔLC3B-II expression between WT and KO myoblasts (*p* = 0.24) (n = 10). (**B**) Western blot images showing p62 protein levels in wildtype (WT) and knockout (KO) primary myoblasts, both under basal (Baf−) and bafilomycin A1-treated conditions (Baf+). a-tubulin was analyzed as loading control. Box plot shows significant difference in Δp62 expression between WT and KO myoblasts (*p* = 0.04) (n = 7). p62 shows increased accumulation in KO cells upon bafilomycin treatment. (**C**) Western blot images showing LC3B-II protein levels in wildtype (WT) and knockout (KO) ESCs, both under basal (Baf−) and bafilomycin A1-treated conditions (Baf+). a-tubulin was analyzed as loading control. Box plot shows no significant difference in ΔLC3B-II expression between WT and KO myoblasts (*p* = 0.18) (n = 6). (**D**) Western blot images showing p62 protein levels in wildtype (WT) and knockout (KO) ESCs, both under basal (Baf−) and bafilomycin A1-treated conditions (Baf+). a-tubulin was analyzed as loading control. Box plot shows significant accumulation of Δp62 expression between WT and KO myoblasts (*p* = 0.003) (n = 5). (**E**) Western blot images showing LC3B-II protein levels in C2C12 and Atoh8-OE myoblasts, both under basal (Baf−) and bafilomycin A1-treated conditions (Baf+). a-tubulin was analyzed as loading control. Box plot shows significant accumulation of ΔLC3B-II in Atoh8-OE samples compared to C2C12 myoblasts (*p* = 0.007) (n = 3). (**F**) Western blot images showing p62 protein levels in C2C12 and Atoh8-OE myoblasts, both under basal (Baf−) and bafilomycin A1-treated conditions (Baf+). a-tubulin was analyzed as loading control. Box plot shows significant accumulation of Δp62 expression between C2C12 and Atoh8-OE myoblasts (*p* = 0.0008) (n = 4).

To further validate the role of Atoh8 in autophagy, we evaluated LC3B-II and p62 expression following treatment with Bafilomycin A1, a lysosomal V-ATPase inhibitor that blocks autophagosome-lysosome fusion [44]. Supporting the previous observations, following 24 h treatment of primary myoblasts with Bafilomycin A1 (10 nM), a non-significant decrement in ΔLC3B-II levels (LC3B-II expression in the presence of Bafilomycin A1 and absence of Bafilomycin A1) (WT vs. KO; *p* = 0.24), and at the same time, a significant increment in Δp62 levels (p62 expression in the presence of Bafilomycin A1 and absence of Bafilomycin A1) was observed (WT vs. KO; *p* = 0.04), suggesting impairment of autophagy at both autophagosome formation and degradation (Figure 5A,B). Similar results were observed in embryonic stem cells following 24 h of treatment with Bafilomycin A1 (10 nM), LC3B-II (WT vs. KO; *p* = 0.18), and p62 (WT vs. KO; *p* = 0.003) (Figure 5C,D). Confirming the role of Atoh8 in autophagy, stable overexpression of Atoh8 in C2C12 myoblasts showed increased accumulation of LC3B-II and p62 following 24 h Bafilomycin A1 (100 nM) treatment, suggesting enhanced autophagy (Figure 5E,F). Overall, these findings corroborate the findings observed in basal conditions.

### 3.6. Overexpression of Atoh8 in C2C12 Cells Alters Mitochondrial and Lysosomal Function

**Figure 6:**
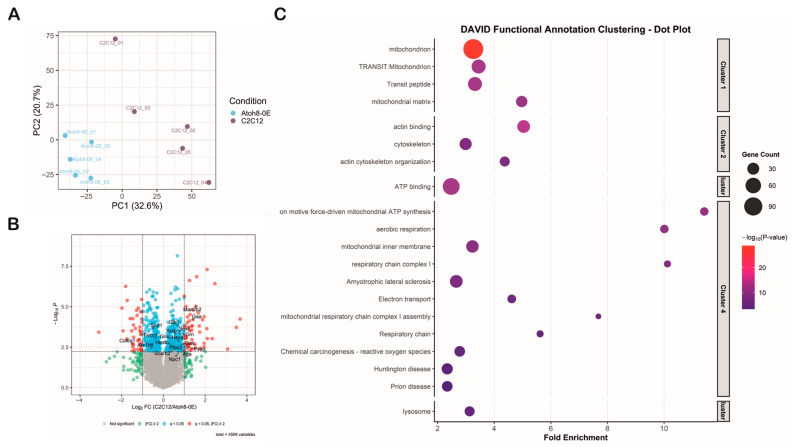
Proteomic profiling shows that Atoh8 contributes to the regulation of mitochondrial and lysosomal function. (**A**) Principal Component Analysis (PCA) demonstrated separation between the C2C12 and Atoh8-OE samples based on their proteomic profiles, indicating distinct molecular signatures for each group. PCA was performed using 4509 consistently quantified protein groups (hereafter referred to as proteins). (**B**) The volcano plot illustrates the results of the differential expression analysis between the C2C12 and Atoh8-OE groups. The *x*-axis represents the log2 fold change (C2C12/Atoh8-OE), while the *y*-axis shows the log10 *p*-value. Proteins that were significantly dysregulated (q < 0.05, fold-change ≥ 2) are shown as red dots. Proteins that were significantly dysregulated (q < 0.05) but with a fold change in less than 2 are depicted as blue dots. Proteins with fold changes less than 2, regardless of significance, are shown in green. Proteins with no significant differences are represented by gray dots. Arrow marks show the position of the protein in the volcano plot. (**C**) DAVID functional annotation clustering dot plot of enriched biological processes and pathways among differentially expressed proteins. The fold enrichment is shown on the *x*-axis, and gene ontology or KEGG terms are listed on the *y*-axis. Dot size corresponds to gene count, while color intensity represents the −log10 *p*-value, with red indicating the most significant terms. The clustered terms highlight mitochondria (Cluster 1), cytoskeleton organization (Cluster 2), ATP binding (Cluster 3), Mitochondrial function, aerobic respiration and neurodegenerative disease-related pathways (Cluster 4), and lysosomal function (Cluster 5) indicating functional categories altered by Atoh8 overexpression.

To investigate the impact of Atoh8 overexpression on autophagic flux, we performed data-independent acquisition (DIA) mass spectrometry-based proteomics on lysates derived from five replicates of C2C12 and Atoh8-Flag overexpressing C2C12 cells (Atoh8-OE). Principal component analysis (PCA) separated the C2C12 and Atoh8-OE samples, suggesting distinct proteomic profiles for each cell type (Figure 6A). In total, 4509 protein groups (hereafter referred to as proteins for better readability) were quantified in all samples, of which 276 (q < 0.05) were significantly downregulated and 257 (q < 0.05) were significantly upregulated in Atoh8-OE samples compared to C2C12 cells (Figure 6B). Differentially expressed proteins were subsequently analyzed using DAVID functional annotation clustering. The top five significantly enriched clusters included mitochondria, aerobic respiration, cytoskeletal organization, ATP binding, and lysosomal function (Figure 6C). Notably, proteins involved in lysosomal function and autophagy regulation exhibited significant dysregulation. Specifically, we observed significant upregulation of lysosomal hydrolases and associated proteins including Npc1, Arsb, Aga, Glb1, Galc, Gns, Gaa, Grn, Hexa, Hexb, Man2b2, Plbd2, Psap, Scarb2, and Snx6 in Atoh8-OE samples compared to C2C12 samples. Conversely, significant downregulation was detected for Fyco1, Akr1b8, Sod1, and Col6a1 (Supplementary Table S1). Many of these proteins are implicated in lysosomal storage disorders resulting in secondary autophagic impairment. Collectively, these proteomic data demonstrate that Atoh8 overexpression induces alterations in lysosomal-autophagic machinery, consistent with regulatory role of Atoh8 on autophagy.

### 3.7. Atoh8 Regulates Transcription of Multiple Genes Involved in Autophagy

**Figure 7:**
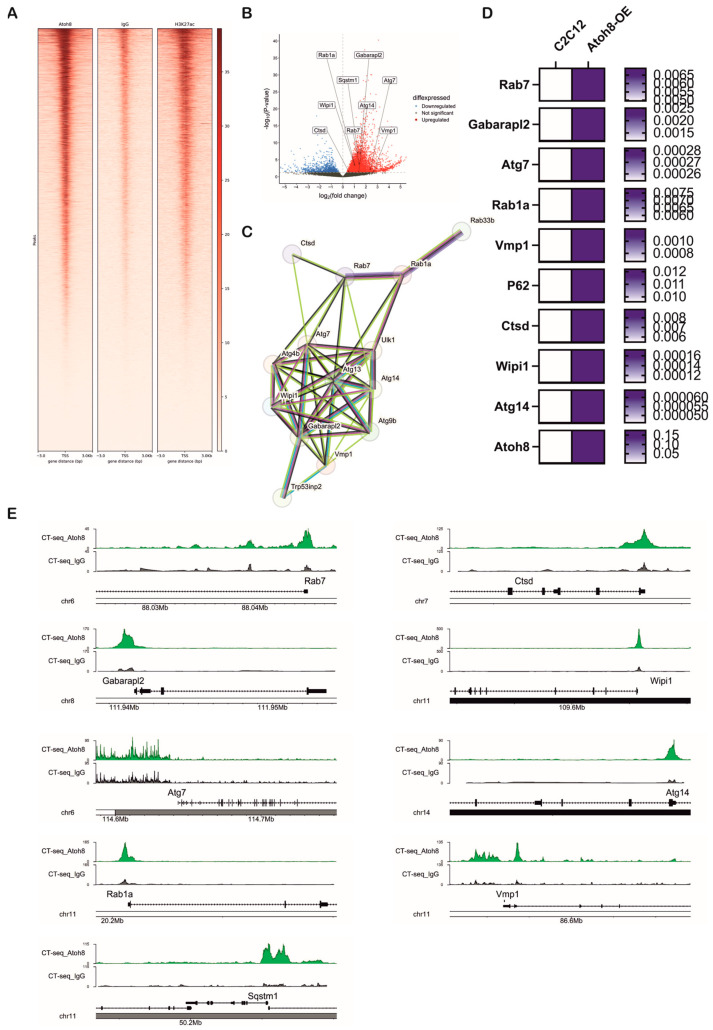
Atoh8 regulates transcription of multiple genes involved in autophagosome assembly. (**A**) Heatmap showing genome-wide enrichment signals for Atoh8, IgG control, and H3K27ac at gene loci, with distance from the gene center (in base pairs) on the *x*-axis and the signal intensity on the *y*-axis. This demonstrates specific binding patterns of Atoh8 compared to controls and active histone mark H3K27ac. (**B**) Volcano plot depicting differential expression of autophagy-associated genes. Genes are plotted by log2 fold-change on the *x*-axis and statistical significance (−log10 *p*-value) on the *y*-axis. Upregulated genes are highlighted in red, downregulated in blue, and non-significant genes in gray. Labeled genes include *Rab1a*, *Gabarapl2*, *Sqstm1*, *Atg7*, *Wipi1*, *Ctsd*, *Rab7* among others, indicating their significant modulation. (**C**) Protein–protein interaction network generated by STRING shows autophagy-related proteins identified from the dataset. The network highlights a complex interconnected cluster suggestive of coordinated regulation. (**D**) Heatmap showing validation of the genes that were upregulated following overexpression of Atoh8 in C2C12 myoblasts. The genes that showed significantly upregulation (*p* < 0.05) in real-time PCR. White color suggests lower mRNA expression whereas blue color represents higher mRNA expression. (**E**) Genome browser tracks comparing Cut-and-Tag enrichment profiles at key autophagy gene loci (*Rab7*, *Gabarapl2*, *Atg7*, *Rab1a*, *Vmp1*, *Sqstm1*, *Ctsd*, *Wipi1*, *Atg14*). Tracks show experimental signal (green) against a control or baseline (gray), and gene structure (black).

We next investigated transcriptional targets of ‘Atoh8’ by subjecting Atoh8-Flag overexpressing C2C12 cells (Atoh8-OE) to Cut&Tag sequencing. We made this choice because of unavailability of a reliable commercial antibody and because of short half-life of Atoh8 protein (approximately 30 min). We performed Cut&Tag sequencing following the culture of cells for 24 h in nutrient rich media. Cut&Tag sequencing revealed chromatin occupancy of Atoh8 at transcriptional start sites of multiple genes that are involved in the regulation of autophagy (*Rab7*, *Gabarapl2*, *Atg7*, *Rab1a*, *Vmp1*, *Sqstm1 (p62)*, *Ctsd*, *Wipi1*, *Atg14*, *Rab33b*, *Ulk1*, *Atg9b*, *Trp53inp2*, *Atg4b*) (Figure 7A,B and Supplementary Table S2). Functional annotation using STRING enriched these proteins to the autophagosome assembly network (Figure 7C). Next, we validated if this binding of Atoh8 at transcriptional start sites of the above-mentioned genes result in the alteration of transcription using real-time PCR. Notably, we observed significant upregulation of transcripts of *Rab7*, *Gabarapl2*, *Atg7*, *Rab1a*, *Vmp1*, *Sqstm1*, *Ctsd*, *Wipi1*, *Atg14* with no significant changes in the transcripts of *Rab33b*, *Ulk1*, *Atg9b*, *Trp53inp2*, *Atg4b* (Figure 7D). The defined peaks of the transcriptional start sites of the validated genes are shown in (Figure 7E). Overall, Cut&Tag sequencing revealed that Atoh8 indeed regulates transcription of genes involved in autophagosome assembly which is in line with the above observations.

## 4. Discussion

Atoh8 belongs to a large superfamily of highly conserved transcription factors, known as bHLH transcription factors. It has been shown to be expressed in various organs during murine development. Studies on murine and human cell types have implicated Atoh8 in the regulation of iron homeostasis and carcinogenesis. In our previous studies, we identified Atoh8 as a regulator of stem cell fate and skeletal muscle differentiation, reporting significant alterations in skeletal muscle fiber types and muscle mass, suggesting metabolic changes in the absence of Atoh8. Overall, we determined that the loss of Atoh8 leads to muscular atrophy. To understand the significance of Atoh8 in metabolism, we first investigated whether Atoh8 is involved in the catabolic process called autophagy, a crucial process that eliminates or recycles intracellular proteins and organelles. First, we analyzed the levels of LC3B-II, an autophagic flux marker involved in autophagosome formation, cargo recognition, tethering, and fusion of autophagosomes with lysosomes in primary myoblasts and mESCs derived from WT (*Atoh8^Flag-tag^*) and KO (*Atoh8^−^^/^^−^*) mice. LC3B-II was quantified after culturing the cells under nutrient-rich conditions for 24 h. We did not observe any significant difference in LC3B-II in primary myoblasts; however, LC3B-II in KO-ESCs was significantly decreased compared to that in WT-ESCs. Furthermore, we have evaluated the LC3B-II/LC3B-I ratio’s and observed that stable loss of Atoh8 results in higher LC3B-II/LC3B-I ratio’s suggesting altered autophagic activity in primary myoblasts and ESCs. To confirm these findings, we further evaluated the autophagy receptor ‘p62’ in primary myoblasts and ESCs. Surprisingly, we observed a significant accumulation of p62 following the knockout of Atoh8 in embryonic stem cells and terminally differentiated primary myoblasts. We further analyzed LC3-GFP puncta in WT and KO primary myoblasts following stable infection with the GFP-LC3-RFP virus, and the data analysis confirmed significantly smaller autophagosomes in KO primary myoblasts, suggesting impaired autophagy. Typically, autophagy impairment results in the accumulation of LC3B-II and p62. Considering the complexity of autophagy regulation, impairment of autophagy can also result in normal LC3B-II or decreased LC3B-II despite the accumulation of p62. Studies have shown that post-translational inactivation of ATG4B, either by reversible oxidation [45,46], phosphorylation [47], S-nitrosylation [48], or ubiquitination [49], inhibits the recycling of LC3B-II, despite the accumulation and impairment of autophagy. Overall, the observations of LC3B-II, LC3B-II/LC3B-I ratio’s, p62, and LC3-GFP puncta analysis suggest that the loss of Atoh8 impairs autophagy.

To validate these findings, we generated a stable C2C12 cell line overexpressing the *Atoh8-Flagtag* sequence. Corroborating the involvement of Atoh8 in autophagy regulation, the stable overexpression of Atoh8 resulted in the reduction in p62 levels, with no change in LC3B-II expression. However, a reduction in LC3B-II/LC3B-I ratio was observed suggesting that Atoh8 overexpression promotes autophagy.

To further confirm the above data, we analyzed TFEB, another bHLH transcription factor and master regulator of autophagy and lysosomal biogenesis [50]. We evaluated total TFEB abundance in primary myoblasts and ESCs. However, we did not observe any change in total TFEB expression following Atoh8 loss. At the same time, the total amount of TFEB in the stable C2C12-Atoh8-OE cells was reduced. TFEB function is tightly governed by phosphorylation, which regulates its subcellular localization. Under nutrient-rich conditions, mTORC1 phosphorylates TFEB and promotes cytoplasmic sequestration via the 14-3-3 protein [51]. During starvation or stress, mTORC1 inhibition results in the dephosphorylation of TFEB via PP2A/calcineurin, enabling TFEB translocation into the nucleus, and leading to the activation of autophagy-lysosomal genes [52]. Given the significance of TFEB subcellular localization, we evaluated the cytoplasmic-to-nuclear ratio of the TFEB protein. In line with LC3B-II and p62 expression, and despite no difference in total TFEB levels, the cytoplasmic-to-nuclear ratio in KO primary myoblasts showed less TFEB presence in the nucleus, supporting either lower or impaired autophagic activity in these cells. In contrast to observations in terminally differentiated primary myoblasts, embryonic stem cells showed increased nuclear localization of TFEB despite impaired autophagy. Previously, mESCs were reported to contain high levels of TFEB at both the mRNA and protein levels. However, in mESCs, TFEB possesses other functions independent of its canonical autophagy-lysosomal biogenesis role. It has been shown that core pluripotent network genes Oct4, Sox2, and Nanog bind to the TFEB promoter and generate a feed-forward loop with pluripotency factors. Consistent with this, we previously observed upregulation of Oct4 and Nanog at the mRNA level in KO-ESCs. In C2C12 cells, despite the downregulation of total TFEB following stable overexpression of Atoh8 in C2C12 cells, no difference was observed in the cytoplasmic to nuclear localization between C2C12 and C2C12-Atoh8-OE cells. Overall, the data observed in primary myoblasts, embryonic stem cells, and C2C12 cells indicate that loss of Atoh8 impairs autophagy, and contribute differential localization of TFEB in response to the functional control of Atoh8 on autophagy. Inhibition of autophagy by Bafilomycin further corroborated these findings, indicating that loss of Atoh8 indeed results in accumulation of p62 and impaired autophagic activity. Conversely, overexpression of Atoh8 in C2C12 cells resulted in enhanced autophagy reinforcing these findings.

Next, we subjected C2C12 and stably Atoh8 overexpressing C2C12 myoblasts (Atoh8-OE) to mass spectrometric analysis. The proteins that were found to be significantly upregulated in Atoh8-OE cells include lysosomal hydrolases and processing enzymes (Man2b2, Gaa, Arsb, Glb1, Gns, Hexa, Hexb, Galc, Aga), trafficking proteins (Snx6, Psap, Scarb2), lysosomal membrane and transporter protein (Npc1), lysosomal phospholipase (Plbd2). The upregulation of lysosomal enzymes (Man2b2, Gaa, Arsb, Glb1, Gns, Hexa, Hexb, Galc, and AgaGA) suggests an enhancement in lysosomal degradative capacity, indicating efficient clearance of substrates, recycling of the lysosomal membrane, and maintenance of lysosomal pH and function [53,54,55,56,57,58,59,60,61]. Similarly, in line with lysosomal enzyme expression, upregulation of trafficking proteins (Snx6, Psap, and Scarb2) and transporter protein (Npc1) also suggests improved delivery of enzymes to lysosomes and export of catabolic products, preventing lysosomal storage [62,63,64,65]. Upregulation of Plbd2 is also in line with increased lysosomal activity [66]. Fyco1 is an autophagy adaptor that links LC3-positive autophagosomes to kinesin motors for transport, and its downregulation might be an adaption to retain autophagosomes and decelerate autophagosome–lysosome fusion, thereby preventing the degradative system from becoming overwhelmed [67]. Col6a1 is not well characterized but emerging evidence suggests a link to autophagy. In addition to this, the downregulation of Sod1 and Akr1b8 suggests an altered oxidative state in Atoh8-OE cells. Overall, altered proteomic profile suggests Atoh8 regulates cellular metabolism and autophagy.

In addition to this, we performed Cut&Tag sequencing on Atoh8-OE cells and identified multiple autophagy related genes to be direct targets of Atoh8. Validation of the identified targets with real time PCR showed upregulation of *Rab7*, *Gabarapl2*, *Atg7*, *Rab1a*, *Vmp1*, *Sqstm1*, *Ctsd*, *Wipi1*, *Atg14* suggesting Atoh8 overexpression results in transcriptional activation of these genes. However, chromatin occupancy further showed binding of Atoh8 to transcriptional state sites of *Rab33b*, *Ulk1*, *Atg9b*, *Trp53inp2*, *Atg4b*. It would be interesting to see if their transcription is activated under stressors such as starvation, hypoxia, etc. Upregulation of *Atg7*, *Vmp1*, *Atg14*, *Wipi1*, and *Rab1a* suggests enhanced autophagosome formation, whereas increased expression of *Sqstm1* (p62) and *Gabarapl2* indicates improved cargo recognition. Upregulation of *Rab7* supports more efficient autophagosome–lysosome fusion, and elevated *Ctsd* reflects enhanced lysosomal degradative capacity [28,68]. Overall, the upregulation of autophagy associated genes in Atoh8-OE cells is line with proteomic profile and expression of LC3B-II and p62 expression which supports the notion that Atoh8 plays a crucial role in the regulation of autophagy.

Here, we further explored whether the impairment of autophagy following either loss or downregulation of Atoh8 could explain any of the observed phenotypes during tissue development, differentiation, and cancer. In our previous study, we reported that KO mice showed reduced muscle mass, which is indicative of muscular atrophy. This finding is supported by evidence linking elevated p62 levels and impaired autophagy to sarcopenia, a condition characterized by progressive loss of muscle mass, strength, and function [69]. Elevated levels of p62 have similarly been observed in cancer cachexia, where they appear to confer a protective effect on oxidative myofibers while providing limited protection to fast glycolytic fibers [70]. Consistent with these observations, our previous study demonstrated a reduction in fast glycolytic fibers, indicative of atrophy, alongside an increase in slow-oxidative and fast-oxidative glycolytic fibers, suggesting a fiber-type composition change in response to increased p62 levels [4]. A study investigating the role of Atoh8 in endochondral bone development revealed a reduction in the zones of proliferation and hypertrophic zones of the growth plate, leading to a decrease in skeletal size in both the axial and appendicular bones. Consistent with these findings, knockout of the essential autophagy genes Atg5 or Atg7 resulted in elevated levels of p62 and a reduction in proliferative and hypertrophic zones, thereby compromising the growth of axial and appendicular bones [71,72,73]. Atoh8 has been reported to be indispensable for placental development. Consistent with this, elevated p62 levels have been documented in conditions such as preeclampsia and restricted placental growth, which are associated with abnormal placentation, compromised decidualization, and vascular remodeling, processess in which Atoh8 was also identified essential for placental development [7,74,75].

In cancers, Atoh8 has often been observed to be downregulated in tumor tissues compared to either normal or adjacent non-tumoral tissues. Cancer types such as nasopharyngeal carcinoma, hepatocellular carcinoma, and non-small cell lung cancer show significant downregulation of Atoh8 expression in tumor tissue. Although complex and context-dependent, there is a strong correlation between autophagy and tumorigenesis. In the early stages, autophagy acts as a tumor suppressor, whereas in the late and advanced stages, it acts as a tumor promoter. In nasopharyngeal carcinomas, 92.5% of tumors exhibit elevated p62 levels, which are associated with distant metastasis [76,77]. Additionally, elevated p62 levels are significantly correlated with poor clinical outcomes [78]. Consistent with this, hepatocellular carcinoma also exhibited elevated levels of p62 in tumor tissues compared to adjacent non-tumor tissues, and these higher levels of p62 were associated with poor prognosis. Increased p62 levels are correlated with the enhanced proliferation, migration, and invasion of HCC cells [79,80]. In non-small cell lung cancer, 37% of patients showed p62 accumulation associated with decreased survival, and at the same time higher levels of p62 expression were also significantly associated with aggressive tumor behavior [81,82].

Altogether, these findings substantiate the hypothesis that loss of Atoh8 impairs autophagy. However, the present study specifically addresses macroautophagy. It would be interesting to investigate whether Atoh8 affects distinct autophagic processes, such as microautophagy, selective or chaperone-mediated autophagy, and mitophagy. Furthermore, it would be valuable to ascertain which altered metabolic pathways contribute to the impairment of autophagy following loss of Atoh8. In conclusion, it is plausible to hypothesize that Atoh8 is a novel upstream regulator of autophagy.

## Data Availability

Data will be made available upon request to the corresponding author following the completion of the peer-review process. The mass spectrometry proteomics data have been deposited to the ProteomeXchange Consortium via the PRIDE [doi: 10.1093/nar/gkae1011] partner repository with the dataset identifier PXD070896. All raw and processed sequencing data are available in the Gene Expression Omnibus repository under accession SubmissionID: SUB15749412, BioProject ID: PRJNA1357290.

## Supplementary Materials

The following supporting information can be downloaded at: https://www.mdpi.com/article/10.3390/cells14241993/s1.
