# Supplementary material for: Loss of Atoh8 Impairs Macroautophagy
Source: Cells. 2025 Dec 15;14(24):1993. doi: 10.3390/cells14241993 (PMC12731601; doi:10.3390/cells14241993)
Supplement: Supplementary file 1 [file cells-14-01993-s001.zip › Supplementary Information.pdf]

Supplementary Information

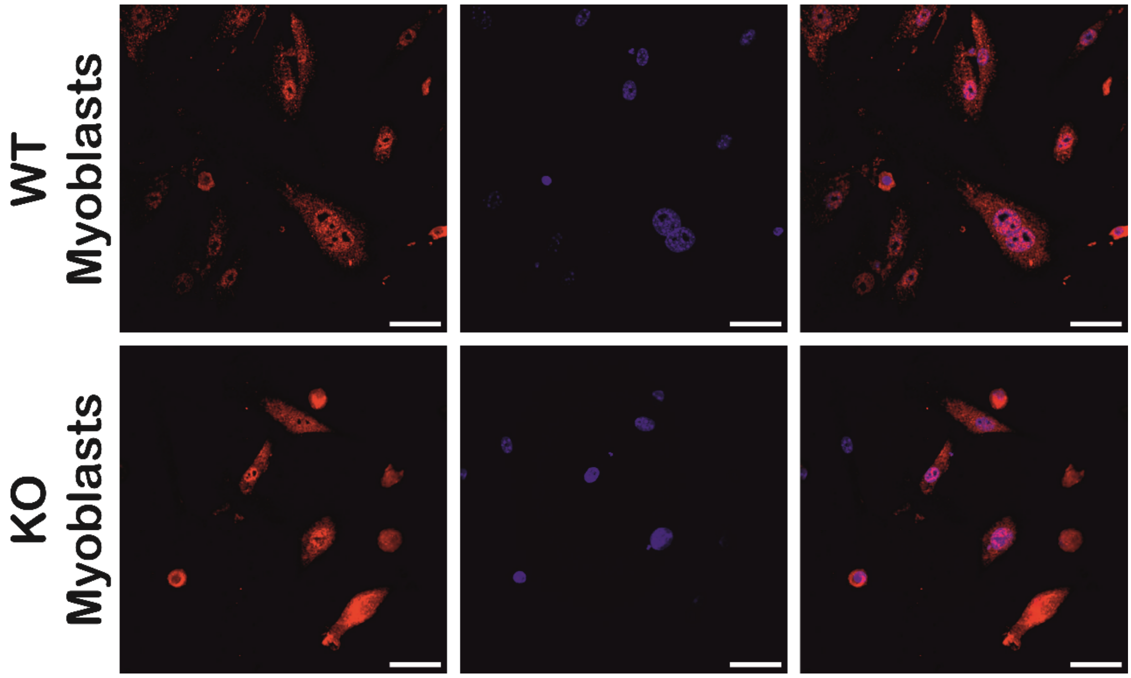

Supplementary Figure S1: TFEB localization in wildtype (WT) and knockout (KO) myoblasts. Representative images depict myoblasts with TFEB staining in red and nuclear counterstaining with DAPI in blue, along with merged images. The subcellular localization of TFEB was quantitatively assessed using a cell profiler pipeline (Human C-N translocation). The scale bars represent 50 µm.

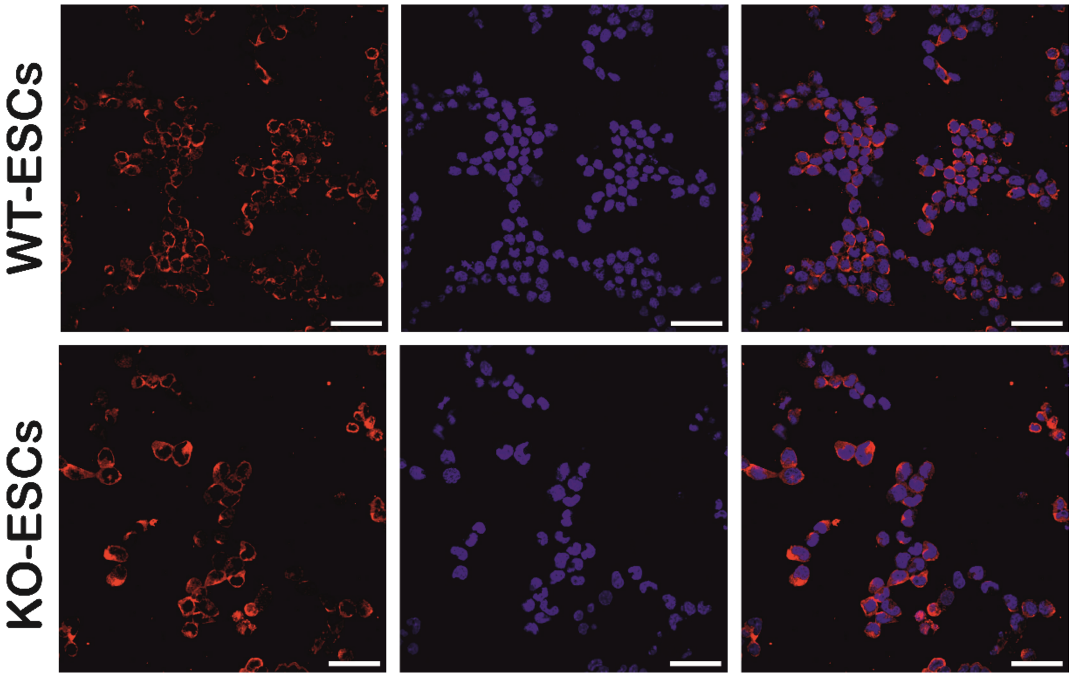

Supplementary Figure S2: TFEB localization in wildtype (WT) and knockout (KO) embryonic stem cells (ESCs). Representative images depict embryonic stem cells (ESCs) with TFEB stained in red and nuclear counterstaining with DAPI in blue, along with merged images. The subcellular localization of TFEB was further quantified using a cell profiler pipeline (Human C-N translocation). The scale bars represent 50 µm.

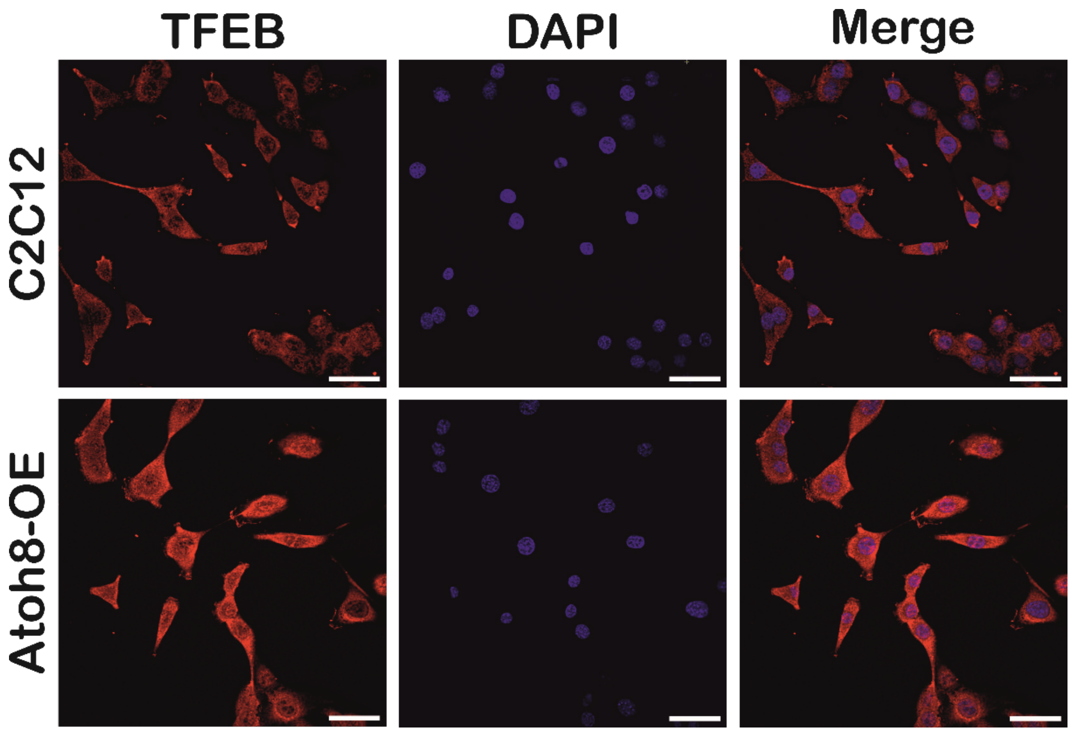

Supplementary Figure S 3: Localization of TFEB in C2C12 (Ctrl) myoblasts and stable C2C12 cells overexpressing Atoh8-Flag (Atoh8-OE). Representative images showing myoblasts with TFEB stained red and nuclei counterstained with DAPI in blue, along with merged images. The subcellular localization of TFEB was further quantified using a cell profiler pipeline (Human C-N translocation). Scale bars indicate 50 µm.

Cell profiler pipeline used quantification of TFEB subcellular localization: (https://cellprofiler.org/examples)

[1] [Images]
To begin creating your project, use the Images module to compile a list of files and/or folders that you want to analyze. You can also specify a set of rules to include only the desired files in your selected folders.

[2] [Metadata]
The Metadata module optionally allows you to extract information describing your images (i.e, metadata) which will be stored along with your measurements. This information can be contained in the file name and/or location, or in an external file.

Here we're going to extract the well location that each image was taken in. We'll also collect the folder the images reside in, so that we can match the illumination correction function with each well next step in NamesAndTypes.

[3] [NamesAndTypes]
The NamesAndTypes module allows you to assign a meaningful name to each image by which other modules will refer to it.

Load the images by matching files in the folder against the unique text pattern for each stain: Channel 1 for protein, Channel 2 for the nuclei image. Also exactly match the text for the illumination correction function file names.

So that you can match each wells images to the single set of illumination correction functions rather than matching the image set by Order weve matched it by Metadata which allows us to instruct CellProfiler that each well will have its own set of 'Nucleaus' and 'RFP' images but that they all use the illumination correction function found in the same folder.

[4] [Groups]
The Groups module optionally allows you to split your list of images into image subsets (groups) which will be processed independently of each other. Examples of groupings include screening batches, microtiter plates, time-lapse movies, etc.

[5] [IdentifyPrimaryObjects]
Identify the nuclei from the nuclear stain image.

[6] [IdentifySecondaryObjects]
Identify the cells by using the nuclei as a "seed" region, then growing outwards until stopped by the image threshold or by a neighbor. The Distance-B method is used to delineate the boundary between neighboring cells.

[7] [IdentifyTertiaryObjects]
Identify the cytoplasm by "subtracting" the nuclei objects from the cell objects.

[8] [MeasureObjectIntensity]
Measure intensity features from nuclei, cell and cytoplasm objects against the corrected images.

[10] [CalculateMath]
Calculate the nuclei-to-cell ratio of the mean protein intensity.

[11] [CalculateMath]
Calculate the cytoplasm-to-cell ratio of the mean protein intensity.

[12] [OverlayOutlines]
Overlay the nucleus and cell outlines on the protein image.

[13] [ExportToSpreadsheet]
Export any measurements to a comma-delimited file (.csv). The measurements made for the nuclei, cell and cytoplasm objects will be saved to separate .csv files, in addition to the per-image .csv's.

List real-time PCR primer sequences:

RT_mRab7_F	AGGCTTGGTGCTACAGGAAAA
RT_mRab7_R	CTTGGCCCGGTCATTCTTGT
RT_mGabarapl2_F	TCGGGCTCTCAGATTGTTGAC
RT_mGabarapl2_R	ATGGCCTTCTCGGAGGGAA
RT_mAtg7_F	TGGCTGCTACTTCTGCAATGATGT
RT_mAtg7_R	CAGGACAGAGACCATCAGCTCCAC
RT_mRab1a_F	TTACTTCTGATTGGCGATTCTGG
RT_mRab1a_R	TGGGCTCCTCTGTAATAACTGG
RT_mVmp1_F	CCAGAGACGCATAGCAATGAG
RT_mVmp1_R	GCAAGGTAATGAGTGGCTGTC
RT_p62_F	AGGATGGGGACTTGGTTGC
RT_p62_R	TCACAGATCACATTGGGGTGC
RT_mCtsd_F	GCTTCCGGTCTTTGACAACCT
RT_mCtsd_R	CACCAAGCATTAGTTCTCCTCC
RT_mWipi1_F	CTCCACGGTGCCAGGATAC
RT_mWipi1_R	AATGCTCACAGGGGGAAACTC
RT_mAtg14_F	GAGGGCCTTTACGTGGCTG
RT_mAtg14_R	AATAGACGAAATCACCGCTCTG
RT_mUlk1_F	AAGTTCGAGTTCTCTCGCAAG
RT_mUlk1_R	CGATGTTTTCGTGCTTTAGTTCC
RT_mAtg4b_F	TATGATACTCTCCGGTTTGCTGA
RT_mAtg4b_R	GTTCCCCCAATAGCTGGAAAG

RT_mRab33b_F	AGACGTGCCTGACTTACCG
RT_mRab33b_R	GTGTCCCACAACTGGATCTTG
RT_mTrp53inp2_F	GCGCCCTCCTTGATGGATG
RT_mTrp53inp2_R	TCCTCCAGCGGATTGCTCT
RT_mAtoh8_F	AGAACTGAACGGCCTCAAGA
RT_mAtoh8_R	GGAGCTTCCAAGTCCAATCG
